# Investigating the associations between personality functioning, cognitive biases, and (non-)perceptive clinical high-risk symptoms of psychosis in the community

**DOI:** 10.1192/j.eurpsy.2024.1812

**Published:** 2025-01-22

**Authors:** Giulia Rinaldi, Stefan Lerch, Frauke Schultze-Lutter, Stefanie Julia Schmidt, Marialuisa Cavelti, Michael Kaess, Chantal Michel

**Affiliations:** 1 University Hospital of Child and Adolescent Psychiatry and Psychotherapy, University of Bern, Bern, Switzerland; 2 Department of Psychiatry and Psychotherapy, Medical Faculty and University Hospital Düsseldorf, Heinrich-Heine-University Düsseldorf, Düsseldorf, Germany; 3 Department of Psychology, Faculty of Psychology, Airlangga University, Surabaya, Indonesia; 4 Department of Clinical Child and Adolescent Psychology, University of Bern, Bern, Switzerland; 5 Department of Child and Adolescent Psychiatry, Centre for Psychosocial Medicine, University Hospital Heidelberg, Heidelberg, Germany

**Keywords:** general population, information processing bias, psychopathology, psychosis risk

## Abstract

**Background:**

Beyond psychosis prediction, clinical high-risk (CHR-P) symptoms show clinical relevance by their association with functional impairments and psychopathology, including personality pathology. Impaired personality functioning is prioritized in recent dimensional personality disorder models (DSM-5, ICD-11), yet underexplored in CHR-P, as are associations with cognitive biases, which early studies indicate as possibly linking CHR-P-symptoms and personality pathology.

**Methods:**

A community sample (*N =* 444, 17–60 years, 61.8% female) was assessed via clinical telephone interview and online questionnaires. Using zero-inflated Poisson models, we explored associations of personality functioning, cognitive biases, current psychopathology, and psychosocial functioning with likelihood and severity of overall CHR-P, as well as perceptive (per-) and non-perceptive (nonper-)CHR-P-symptoms distinctly.

**Results:**

Higher nonper-CHR-P-symptom likelihood was associated with more impaired personality functioning and psychosocial functioning, while more severe cognitive biases were associated with higher CHR-P- and per-CHR-P-symptom likelihood, alongside higher CHR-P- and nonper-CHR-P-symptom severity. Further, more axis-I diagnoses were linked to higher CHR-P-, per-CHR-P-, and nonper-CHR-P-symptom likelihood, and younger age to higher CHR-P- and per-CHR-P-symptom severity, with CHR-P-symptom severity appearing higher in females. In an exploratory analysis, personality functioning elements identity and self-direction, and cognitive biases dichotomous thinking, emotional reasoning, and catastrophizing, respectively, showed multifaceted associations with nonper-CHR-P-symptom likelihood and overall CHR-P-symptom expression.

**Conclusions:**

Our study supports the association of CHR-P-symptoms with multiple mental health factors. Findings suggest intricate associations between personality functioning impairments and cognitive biases with CHR-P-symptom expression in non-help-seeking populations, possibly contributing to different per-CHR-P- and nonper-CHR-P-symptom expression patterns. Therefore, they should be targeted in future longitudinal studies, aiming at better understanding CHR-P-manifestations to inform preventive intervention.

## Introduction

Within the internationally established clinical high risk for psychosis (CHR-P) approach for early risk detection and indicated prevention of first-episode psychosis, risk criteria are primarily identified by presence, time, and severity of CHR-P-symptoms [1]. To define a CHR-P state, two sets of criteria are mainly used: ultra-high risk (UHR) and basic symptoms criteria [2, 3]. Basic symptoms are self-experienced subclinical disturbances in thinking, speech, and perception that patients immediately recognize as disturbances of their own mental processes and are therefore distinct from both UHR-relevant symptoms (i.e., attenuated or brief intermittent psychotic symptoms) and more persistent frank psychotic symptoms [4]. Further highlighting the complexity of these manifestations, perceptive (per; e.g., perceptual basic symptoms, hallucinations) and non-perceptive (nonper; e.g., cognitive basic symptoms, delusions) CHR-P-symptoms exhibit meaningful differences in prevalence, expression, outcome, and clinical significance [5–8]. Specifically, per-CHR-P-symptoms are more common, but less clinically relevant, in children and adolescents, with related psychological and functional burden increasing as they stabilize by age 18 [6, 9]. In contrast, prevalence of nonper-CHR-P-symptoms is more consistent across age groups, and they show earlier clinical significance, particularly in late adolescence, due to their stronger link to functional impairments and psychiatric comorbidities [6, 10]. These differences suggest that per-CHR-P-symptoms reflect earlier-stage maturation, while nonper-CHR-P-symptoms align with later-stage processes [10]. While the CHR-P state remains associated with an increased risk of psychotic disorders, recent declines in conversion rates to psychosis, alongside high rates of comorbidity with non-psychotic psychopathology, have raised questions regarding its specificity [11–14]. Simultaneously, this evidence, coupled with associations between CHR-P-symptoms and impairments in neurocognitive and psychosocial functioning, underscores the burden associated with the CHR-P state, criteria, and symptoms, independently of conversion to manifest psychosis [15–19]. As psychotic disorders are increasingly conceptualized as existing along a continuum, from normativity to more severe psychopathology (DSM-5; [20]), and this hypothesis is gaining empirical support [16], focus is shifting toward the role of the CHR-P state, criteria, and symptoms in broader mental health contexts, and their mapping onto dimensional, symptom-driven models of psychopathology [16, 21, 22–25]. These efforts include investigation of the associations between CHR-P-symptoms and other severe mental disorders or symptom dimensions and may ultimately contribute to a better understanding of the full spectrum of mental health, with potential applications in both clinical and community settings [10, 24, 26, 27]. Specifically, understanding CHR-P-symptoms within the community can provide valuable insights into the psychosis continuum, where UHR- and basic symptoms occur at varying frequencies and levels of severity [10, 28]. In this context, personality pathology emerges as a factor of particular interest, as evidence has consistently linked it to psychosis development and the psychosis continuum [29–31]. Both clinically significant personality traits (e.g., borderline, schizoid, schizotypal, avoidant) [31, 32] and expression patterns of personality domains [29] have been associated with psychotic disorders and CHR-P. Among several models of personality structure, research predominantly features the Big Five Model [29, 30, 33]. Studies have found that high neuroticism and low extraversion predict schizophrenia onset [29, 34, 35], and patients with first-episode psychosis additionally show higher openness and agreeableness, but lower extraversion and conscientiousness than controls [36]. Further, openness has been associated particularly with subclinical psychotic symptoms and psychotic proneness [30, 37]. Moreover, in patients with psychosis, frequent comorbidity with avoidant, schizoid, paranoid and schizotypal personality disorders has been reported [30], and studies involving CHR-P samples have consistently found a high prevalence (on average 39.4%) of personality disorders, most frequently schizotypal and borderline [38]. Yet, despite growing evidence of associations between psychosis (risk) and personality pathology, the direction of any causal associations remains unclear, and evidence on the role of specific personality disorders and traits in CHR-P and conversion to psychosis is inconclusive [38–40]. Therefore, recent literature suggests that, rather than specific traits or personality disorders, the essential and most impairing features of personality pathology – that is, disturbances in the self and interpersonal domains [36] – might underpin its association with psychosis and the CHR-P state [39, 40]. This proposition aligns with the Alternative Model of Personality Disorders in DSM-5 (AMPD; [20]), where moderate or greater (≥Level 2 on a 0–4 scale) impairments along two dimensions of overarching personality functioning, that is, self- and interpersonal functioning, constitute the essential diagnostic feature (Criterion A), complemented by maladaptive personality traits (Criterion B). Self-functioning captures identity and self-direction, encompassing a stable, coherent experience of the self as well as effective emotional regulation, self-reflection, and directed behavior [20]. In contrast, interpersonal functioning refers to interactive aspects of personality functioning, including empathy toward others as well as desire and capacity for intimacy [20]. These features are central to AMPD-personality pathology as they effectively distinguish personality disorders from both normative personality and other psychopathology (e.g., [41]). Further highlighting their relevance, research indicated that personality functioning impairment predicts important negative outcomes such as impaired psychosocial functioning, for example, more accurately than categorial personality disorder (PD) diagnoses, and might address some well-known shortcomings of categorial conceptualizations, including accounting for comorbidity among personality disorders [41, 42]. Thus, in recognition of their clinical utility, dimensional approaches are being embraced more broadly [43], as further exemplified by the new ICD-11, also prioritizing personality functioning impairments in personality disorder diagnoses [44].

This conceptualization is relevant to the associations between personality pathology and psychosis risk because disruptions affecting the self and interpersonal relationships have also been observed along the psychosis continuum [40, 45–47]: moving on from its milder end toward manifest psychosis, progressively permeable self-other boundaries, self-disturbances, and gradual disruptions of narrative identity emerge, as well as impairment in interpersonal functioning [40, 45, 46, 48, 49]. However, research on personality functioning, especially within CHR-P, is still limited [30, 40].

Among factors proposed in literature as potentially underlying the association between psychotic/CHR-P-symptoms and personality pathology, cognitive biases often associated with psychosis emerge as an interesting candidate [47, 50–52]. Indeed, these particular cognitive biases, that is, stable and pervasive systematic distortions in information processing which were initially conceptualized as psychosis-specific, were later also associated with borderline personality disorder, independently from psychiatric comorbidity or a history of psychotic symptoms [51, 53–55]. Moreover, cognitive biases originally linked to psychosis were associated with greater frequency and severity of CHR-P-symptoms in community samples, as well as personality traits and disorders implicated in CHR-P-symptom development [50, 52, 56]. One possible explanation for these associations is that cognitive biases function as the operational component of personality features, actively shaping and sustaining maladaptive beliefs which predispose individuals to psychopathology and psychosis risk [52]. Yet, despite growing evidence suggesting an association of cognitive biases with both personality pathology and CHR-P, existing research has not yet, to our knowledge, explored them together with either CHR-P or a specific focus on personality functioning [47, 50]. Therefore, we explored the associations of personality functioning impairment and cognitive biases with the presence and expression of CHR-P-symptoms in the community. More precisely, our primary research question investigated whether overall personality functioning impairment and cognitive biases were associated with the occurrence and severity of CHR-P-symptoms, controlling for associations with current psychopathology and socio-occupational functioning, as these factors are known to relate to CHR-P-symptom presentation [17, 57]. In a second step, consistent with the AMPD framework (Supplementary Materials, eTable 2), whenever personality functioning impairment (i.e., Criterion A) was significantly associated with CHR-P-symptom occurrence or severity, we further examined maladaptive personality traits (i.e., Criterion B) for associations with CHR-P-symptom occurrence and severity. Finally, to address possible differences between CHR-P-symptom subtypes, we additionally tested these associations on per-CHR-P- and nonper-CHR-P-symptoms separately, drawing on the evidence of differences in their manifestation, trajectory, and underlying mechanisms [5, 6, 8].

## Methods

### Recruitment and procedures

Our analyses involved cross-sectional data from an initial sample of 450 participants (age 17–60 years) who had completed the add-on questionnaires to the second follow-up (ethics ID: 2020–02856) of the “Bern Epidemiological At-Risk” (*N =* 418) and the “Bi-national Evaluation of At-Risk Symptoms in Children and Adolescents” (*N =* 32) community studies by November 2023 (see Supplementary Materials, eFigure 1 for details on the current sample; [5, 58, 59]). Requirements for participation in the add-on study were provision of *ad hoc* informed consent, fluency in German, and no history of psychosis. Data were collected via a main clinical interview conducted via telephone (duration: 45–90 minutes) and add-on self-report questionnaires, filled out online (unless participants expressly requested a paper copy, which they sent back via mail after completion). All data were recorded on REDCap electronic data capture tools (https://projectredcap.org) hosted at the University of Bern [60]. Results of a previous feasibility study supported the reliability of the telephone assessment, showing 78–100% concordance rates with face-to-face interviews [61]. Further information on study procedures and recruitment can be found in eText 1.

### Assessments

#### CHR-P-symptoms

Presence of CHR-P-symptoms was evaluated during the telephone assessment with (i) the Structured Interview for Psychosis-Risk Syndromes (SIPS; [62]), assessing positive UHR-symptoms, and (ii) the Schizophrenia Proneness Instrument, in its Adult (SPI-A; [63]) and Child and Youth (SPI-CY; [64]) versions, assessing basic symptoms. Evidence indicated excellent median inter-rater reliability (*k* = 0.89), as well as strong predictive, convergent, and discriminant validity for the SIPS [65], good inter-rater reliability and discriminant validity for SPI-A [66] and SPI-CY [67].

SIPS-positive scales and SPI-A/CY-items are rated on a 0–6 scale according to their severity and frequency, respectively. We did not consider CHR-P criteria (Supplementary Materials, eTable 1), both because conversion was not our focus, and to increase power, as, consistently with data from earlier assessment times (e.g., [68]), an absolute minority of our sample met the criteria (0.22% for UHR, 2.67% for COPER, and 0.67% for COGDIS).

Next, we created three composite sum-scores by summing individual item scores (range: 0–6). First, we calculated: (i) a per-CHR-P-sum-score (0–18), by adding scores from the SIPS-P4 item and the two SPI-A/-CY items assessing perceptual abnormalities/hallucinations; and (ii) a nonper-CHR-P-sum-score (0–96), by summing scores from all remaining items (Supplementary Materials, eTable 1). These two scores were then added to obtain (iii) an overall CHR-P-sum-score (0–114).

#### Personality pathology

We evaluated severity of personality functioning impairment (Criterion A, AMPD) on the Level of Personality Functioning Scale-Brief Form 2.0 (LPFS-BF 2.0; [69]), which showed good reliability and construct validity [70]. Each item measures impaired functioning (0–3) in one of 12 facets, capturing impairments in identity, self-direction, empathy, and intimacy (i.e., personality functioning-elements), and providing an overall sum-score.

Further, we assessed maladaptive personality traits (Criterion B, AMPD) with the Personality Inventory DSM-5 (PID-5-BF; [71]), wherein scores (0–3) in 25 items are clustered in five higher-order personality trait domains (i.e., negative affect, detachment, antagonism, disinhibition, and psychoticism), and used to calculate an average total score. Evidence on this instrument showed medium to good convergence and discriminant validity [72].

Both instruments were filled out online.

#### Cognitive biases

Cognitive biases were evaluated with the Cognitive Biases Questionnaire for psychosis (CBQp; [73]), also administered online. The questionnaire assesses five cognitive distortions (i.e., jumping to conclusions, intentionalizing, catastrophizing, emotional reasoning, dichotomous thinking) of clinical relevance and high frequency in psychosis, using five subscales. For each of 30 vignettes describing everyday events, respondents choose the most likely between three alternative cognitive responses, illustrating absence (scored 1), possible (2), or likely presence (3) of interpretation bias. Then, summing item-scores resulted in an overall sum-score (30–90). The CBQp showed good internal consistency and excellent test–retest reliability, with its use of indirect questioning of cognitive biases, rather than their direct assessment and labeling, effectively countering the risk of report bias [73].

#### Psychopathology

We assessed current Axis I-psychopathology during the telephone interview with the Mini-International Neuropsychiatric Interview [74], based on DSM-IV psychiatric diagnoses and demonstrating acceptable to high accuracy as well as overall good psychometric properties [75–77]. A score of 1 on the scale assessing each disorder indicated its presence and contributed to the psychopathology sum-score (0–22) reflecting the number of current psychiatric diagnoses.

#### Socio-occupational functioning and sociodemographic variables

Functioning was assessed with the Social and Occupational Functioning Assessment Scale (SOFAS; 0–100; [75]), a widespread measure of functioning often chosen for its simplicity [78]. Further, we included sex, age, and education level (International Standard Classification of Education or ISCED [79]) as covariates in our models. This data was obtained during the main telephone assessment.

### Statistical analyses

Data analysis was conducted in RStudio version 4.3.2., using the *stats* and *pscl* packages.

After listwise deletion of six observations with missing values, we z-standardized the sum-scores evaluating personality functioning impairment, cognitive biases, PID-5, current psychopathology, and socio-occupational functioning, as well as each subscale of the first three. Next, in order to account for overrepresentation of zeros in our outcome variables (i.e., CHR-P, per-CHR-P, and nonper-CHR-P-symptoms), we built three zero-inflated Poisson (ZIP) models [80–82]. ZIP models are particularly well-suited to modeling outcomes that are infrequent yet potentially of substantial relevance, making them appropriate for exploring factors contributing to CHR-P symptomatology in the community [81]. While traditional count models (e.g., Poisson regression) would likely lead to biased interpretation of this highly skewed data, ZIP models account for the existence of two distinct underlying processes suggested by the skewed distribution: one determining the likelihood of zero instances of the outcome and the other modeling the count of non-zero instances [81]. In our study, each ZIP model comprised (i) a zero-inflation model, describing how predictors and covariates influenced the likelihood of the outcome variable being zero on a binary distribution, (ii) a count model, describing how predictors and covariates influenced the actual value of the outcome variable on a Poisson distribution. Moreover, each model included: (i) the sum-scores for the two main predictors – personality functioning impairment and cognitive biases – and the control variables including current psychopathology and socio-occupational functioning; (ii) the covariates age, sex, and education level; (iii) the per-CHR-P-, nonper-CHR-P-, and CHR-P-sum-scores as the respective outcome. Then, in the final sample (*N =* 444), we tested each ZIP model against an equivalent Poisson model, wherein a lower Akaike Information Criterion indicated better data fit [83]. In models where personality functioning was a significant predictor (p < .05), we included the PID-5-sum-score (maladaptive personality traits) as an additional predictor, and ran a Likelihood Ratio test with the *lmtest* R-package, wherein significance (p < .05) indicated improved model fit. In models where personality functioning or cognitive biases were significant predictors (p < .05), we reiteratively replaced them with each of their subscales to analyze their individual contribution, thus testing 19 additional models. Our choice of this procedure, and against simultaneous inclusion of all subscales in one model, was made to avoid multicollinearity, which can arise from high correlations between subscales of an instrument or between instruments measuring related constructs (e.g., LPFS and PID-5, both measuring features of personality). Results of this explorative analysis should be interpreted with caution.

We did not correct for multiple testing in light of (i) the limited number of statistical tests involving the two main predictors (personality functioning and cognitive biases) across three models (six in total), (ii) the correlation between our three outcomes (CHR-P-, per-, and nonper-CHR-P-symptoms), and (iii) the overall exploratory nature of our calculations, which did not involve exact hypotheses on associations between the main variables. All together, these factors determined a limited risk of Type I error, which should most critically be controlled for via multiple testing correction when conducting several comparisons between independent data or in confirmatory designs [84, 85]. In our design, this was weighed against the greater risk of obtaining excessively conservative effect estimates by adjusting p-values [86, 87], and the procedure was considered inappropriate.

## Results

### Sample characteristics

**Figure 1.**
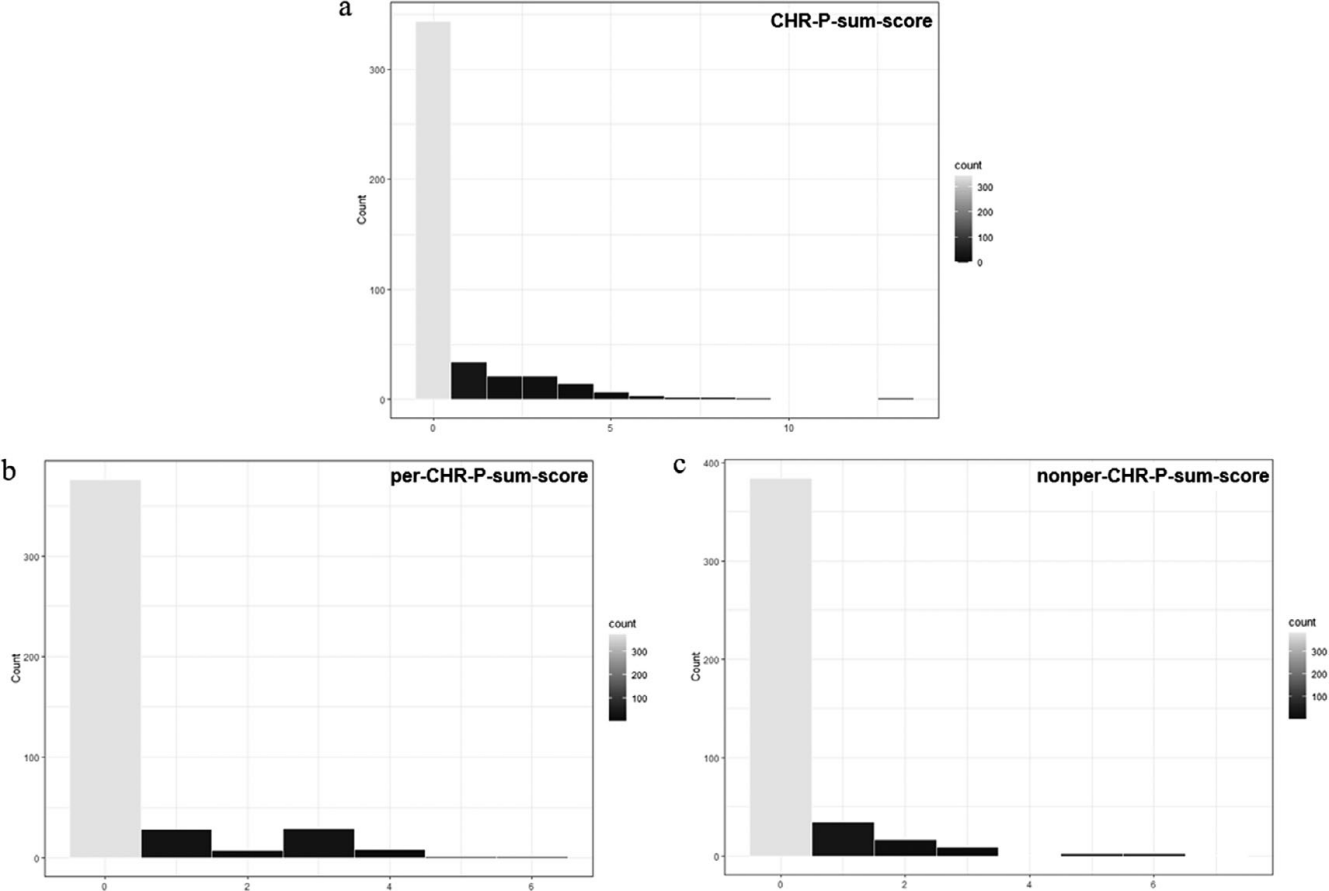
Sample distribution of CHR-P (Figure 1a), per-CHR-P (Figure 1b), and nonper-CHR-P (Figure 1c) sum-scores. On the *x*-axis: sum-score value; on the *y*-axis: number of participants (“count”) presenting with each sum-score value.

Our sample comprised a majority of adult (99.77%), female, highly educated, functionally unimpaired (SOFAS > 70; 94.4%) participants (Table 1). As expected in a community sample, most participants showed no current axis-I disorders, personality functioning impairment was below clinical levels, maladaptive personality traits were below reported elevation cut-offs (Table 1, Supplementary Materials, eTable 2; e.g., [88]), and for most participants the CHR-P- (76.44%), per-CHR-P- (83.56%), and nonper-CHR-P-sum-scores (85.33%) were zero (Figure 1).

**Table 1.** Sample characteristics (*N* = 450)

		n	%
Age (mean ± SD, median, range)		39.38 ± 8.56, 42, 17–60	
Sex (female)		278	61.78
Highest professional education (ISCED level)^a^			
Early childhood education (ISCED 0)		0	0
Primary school or school for special needs (ISCED 1)		0	0
Secondary school (ISCED 2)		6	1.33
High school (ISCED 3.4)		6	1.33
High school-level professional education (ISCED 3.5)		13	2.89
Post-secondary non-tertiary education (ISCED 4)		6	1.33
Short cycle tertiary education, bachelor or master (ISCED 5)		405	90.00
Doctoral (ISCED 6)		12	2.67
SOFAS score (mean ± SD, median, range)		84.6 ± 7.81, 88, 47–95	
Current axis-I disorders, sum-score (mean ± SD, median, range)		0.1 ± 0.38, 0, 0–3	
Current CHR-P-symptoms, sum-score (mean ± SD, median, range)		0.67 ± 1.56, 0, 0–13	
Current per-CHR-P-symptoms, sum-score (mean ± SD, median, range)		0.38 ± 0.99, 0, 0–6	
Current nonper-CHR-P-symptoms, sum-score (mean ± SD, median, range)		0.28 ± 0.86, 0, 0–7	
LPFS 2.0-BF, sum-score (mean ± SD, median, range)^b^		0.68 ± 0.42, 0.67, 0–2.08	
Identity (mean ± SD, median, range)^a^		0.73 ± 0.62, 0.67, 0–2.67	
Self-direction (mean ± SD, median, range)^c^		0.74 ± 0.58, 0.67, 0–3	
Empathy (mean ± SD, median, range)^c^		0.70 ± 0.48, 0.67, 0–2.33	
Intimacy (mean ± SD, median, range)		0.54 ± 0.52, 0.33, 0–2.33	
CBQp sum-score (mean ± SD, median, range)^a^		37.65 ± 3.68, 37, 31–55	
Intentionalizing (mean ± SD, median, range)^c^		7.08 ± 0.91, 7, 6–11	
Catastrophizing (mean ± SD, median, range)^a^		7.43 ± 1.18, 7, 6–13	
Dichotomous thinking (mean ± SD, median, range)		6.73 ± 0.97, 6, 6–13	
Jumping to conclusions (mean ± SD, median, range)		8.73 ± 1.34, 9, 6–15	
Emotional reasoning (mean ± SD, median, range)^a^		7.68 ± 1.43, 7, 6–14	
PID–5 BF (mean ± SD, median, range)		0.43 ± 0.26, 0.40, 0–1.24	
Negative affectivity (mean ± SD, median, range)		0.84 ± 0.54, 0.80, 0–2.60	
Detachment (mean ± SD, median, range)		0.51 ± 0.49, 0.40, 0–2.40	
Antagonism (mean ± SD, median, range)		0.28 ± 0.33, 0.20, 0–1.80	
Disinhibition (mean ± SD, median, range)		0.53 ± 0.46, 0.40, 0–2.60	
Psychoticism (mean ± SD, median, range)		0.50 ± 0.46, 0.40, 0–2.20	

Abbreviations: SOFAS, Social and Occupational Functioning Assessment Scale; CHR-P, clinical high-risk of psychosis; per-CHR-P, perceptive CHR-P; nonper-CHR-P, non-perceptive CHR-P; LPFS-BF 2.0, Level of Personality Functioning Scale-Brief Form 2.0; CBQp, Cognitive Biases Questionnaire; PID-5-BF: Personality Inventory DSM-5 Brief Form.

a Data from two participants (0.44%) were missing.

b Data from three participants (0.67%) were missing.

c Data from one participant (0.22%) were missing.

### ZIP models

When compared by data fit, each ZIP model outperformed its equivalent Poisson model (Supplementary Materials, eTable 3) and was therefore retained for further analyses.

#### CHR-P-symptoms

**Figure 2.**
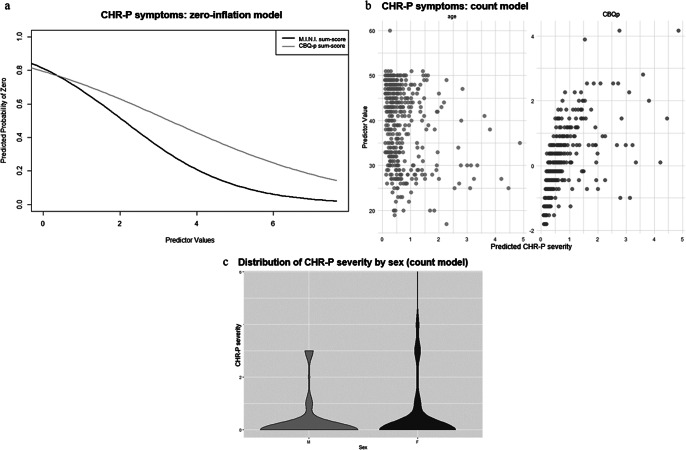
ZIP model results for CHR-P-symptoms. Figure 2a: Zero-inflation model. The *x*-axis shows values of the significant predictor, control variable, or covariate, while the *y*-axis shows the probability of CHR-P-symptoms being zero (e.g., the higher the CBQp-sum-score, indicating more severe cognitive biases, the lower the probability of CHR-P-symptoms being zero). Figure 2b: Count model. The *x*-axis shows predicted CHR-P-symptom severity, while the *y*-axis shows values of the significant predictor, control variable, or covariate (e.g., the younger the age, the higher the predicted CHR-P-symptom severity; the higher the CBQp-sum-score, indicating more severe cognitive biases, the higher the predicted CHR-P-symptom severity). Figure 2c: Count model. The x-axis organizes the data by the significant categorial covariate sex, while the *y*-axis shows predicted CHR-P-symptom severity. Females (F) tend to have a broader distribution of CHR-P-symptom severity, with higher participant density at both lower and higher CHR-P-symptom severity levels, compared to males (M).

In the zero-inflation model, more current axis-I diagnoses (γ = −0.69 ± 0.19, p < .001) and more severe cognitive biases (γ = −0.41 ± 0.15, p = 0.006) were associated with a lower likelihood of the CHR-P-sum-score being 0 (Figure 2a). Additionally, younger age (β = −0.03 ± 0.01, p < .001), female sex (β = 0.32 ± 0.16, p = 0.045), and more severe cognitive biases (β = 0.20 ± 0.07, p = 0.005) were associated with higher CHR-P-sum-scores in the count model (Figure 2b and c). personality functioning was not a significant predictor of either CHR-P-symptom likelihood or severity (Supplementary Materials, eTable 4).

In the exploratory analyses examining individual cognitive biases, more severe catastrophizing (γ = −0.37 ± 0.15, p = .01), dichotomous thinking (γ = −0.27 ± 0.13, p = .04), and emotional reasoning (γ = −0.30 ± 0.13, p = .02) were associated, in their respective zero-inflation models, with lower likelihood of CHR-P-sum-scores being 0. Additionally, in the corresponding count models, more severe dichotomous thinking (β = 0.11 ± 0.05, p = .03) and emotional reasoning (β = 0.21 ± 0.06, p < .001) were associated with higher CHR-P-sum-scores.

#### Perceptive CHR-P-symptoms

**Figure 3.**
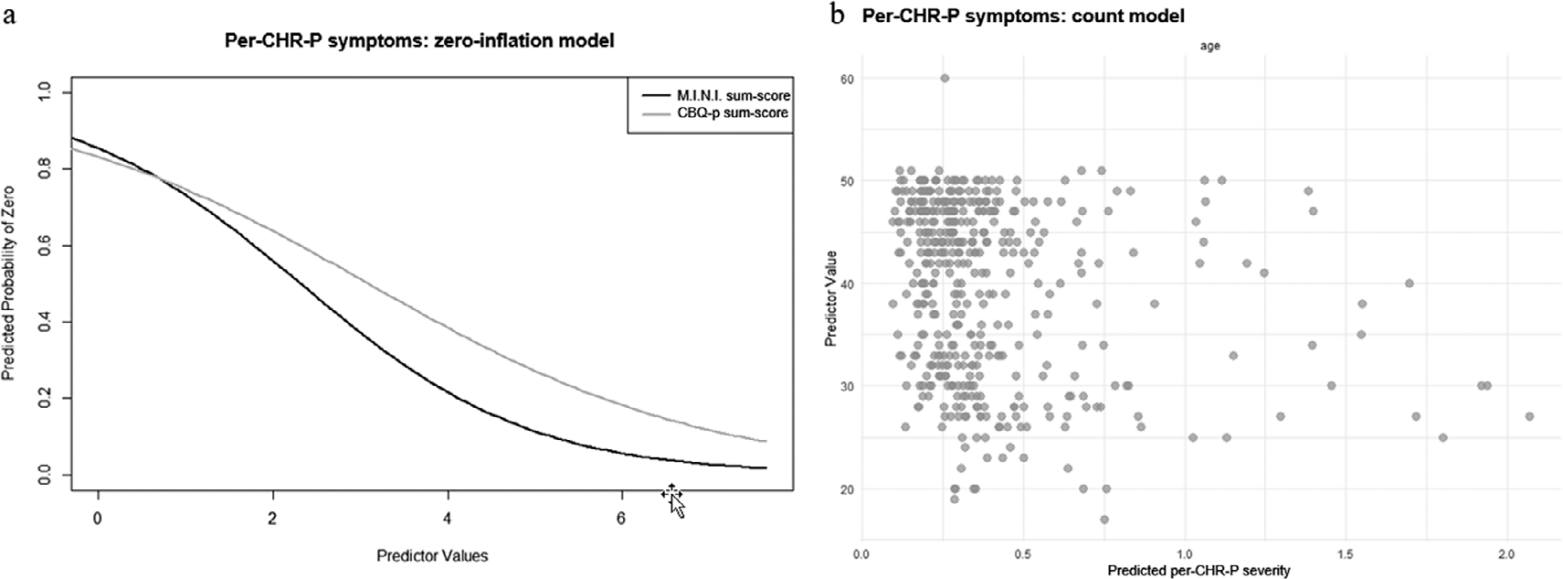
ZIP model results for per-CHR-P-symptoms. Figure 3a: Zero-inflation model. The *x*-axis shows values of the significant predictor, control variable, or covariate, while the *y*-axis shows the probability of per-CHR-P-symptoms being zero (e.g., the higher the CBQp-sum-score, indicating more severe cognitive biases, the lower the probability of CHR-P-symptoms being zero). Figure 3b: Count model. The *x*-axis shows predicted per-CHR-P-symptom severity, while the *y*-axis shows values of the significant predictor, control variable, or covariate (e.g., the younger the age, the higher the predicted CHR-P-symptom severity).

In the zero-inflation model considering only per-CHR-P-symptoms, more current axis-I diagnoses (γ = −0.76 ± 0.18, p < .001) and more severe cognitive biases (γ = −0.52 ± 0.18, p = .003) were associated with lower likelihood of the outcome value being 0 (Figure 3a). In the count model, only younger age was associated with higher per-CHR-P-sum-scores (β = −0.02 ± 0.01, p = .03) (Figure 3b). Personality functioning did not significantly predict either per-CHR-P-symptom likelihood or severity (Supplementary Materials, eTable 5).

As for individual cognitive biases, more severe dichotomous thinking (γ = −0.31 ± 0.14, p = .03) and emotional reasoning (γ = −0.40 ± 0.15, p = .008) were associated – in their respective zero-inflation models – with lower likelihood of per-CHR-P-sum-scores being 0. In the count model, intentionalizing and per-CHR-P-sum-scores were negatively correlated (β = −0.20 ± 0.10, p < .04).

#### Non-perceptive, delusional, or cognitive CHR-P-symptoms

**Figure 4.**
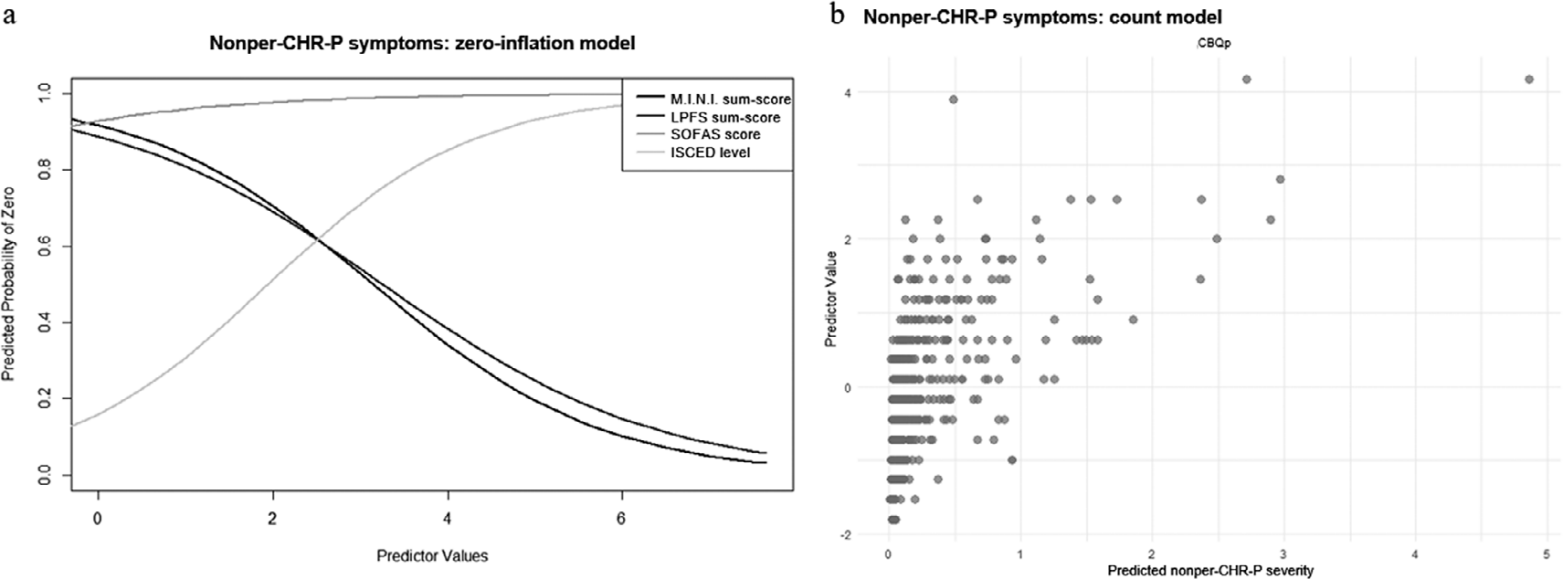
ZIP model results for nonper-CHR-P-symptoms. Figure 4a: Zero-inflation model. The *x*-axis shows values of the significant predictor, control variable, or covariate, while the *y*-axis shows the probability of nonper-CHR-P-symptoms being zero (e.g., the higher the SOFAS-sum-score, indicating higher socio-occupational functioning, the higher the probability of nonper-CHR-P-symptoms being zero; the higher the LPFS-sum-score, indicating higher personality functioning impairment, the lower the probability of nonper-CHR-P-symptoms being zero). Figure 4b: Count model. The *x*-axis shows predicted nonper-CHR-P-symptom severity, while the *y*-axis shows values of the significant predictor, control variable, or covariate (e.g., the higher the CBQp-sum-score, indicating more severe cognitive biases, the higher the predicted nonper-CHR-P-symptom severity).

In the zero-inflation model of nonper-CHR-P-symptoms, more impaired personality functioning (γ = −0.64 ± 0.26, p = .02) and more current axis-I diagnoses (γ = −0.76 ± 0.28, p = .007) were associated with lower, while higher socio-occupational functioning (γ = 0.61 ± 0.31, p = .48) and education level (γ = 0.85 ± 0.40, p = .03) with higher likelihood of having an outcome score of 0 (Figure 4a). Moreover, in the count model, more severe cognitive biases were associated with higher nonper-CHR-P-sum-scores (β = 0.43 ± 0.11, p < .001) (Figure 4b; see Supplementary Materials, eTable 6 for results including non-significant predictors).

Since personality functioning impairment was a significant predictor in this model, we included maladaptive personality traits as an additional predictor and compared the two models via a Likelihood Ratio test, which was non-significant (p = .13; Supplementary Materials, eTable 7), indicating that the new model did not improve fit to our data. Thus, it was discarded.

Finally, examining the impact of individual cognitive biases and personality functioning elements, we found that more pronounced catastrophizing (γ = −0.48 ± 0.21, p = .02), identity impairments (γ = −0.76 ± 0.26, p = .003), and self-direction impairments (γ = −0.56 ± 0.22, p = .009) were associated with lower likelihood of nonper-CHR-P-sum-scores being 0 in the relevant zero-inflation models. In the corresponding count models, more severe intentionalizing (β = 0.34 ± 0.10, p < .001), dichotomous thinking (β = 0.36 ± 0.09, p < .001), and emotional reasoning (β = 0.30 ± 0.09, p < .001) were linked to higher nonper-CHR-sum-scores, while higher impairments in identity (β = −0.26 ± 0.11, p = .03) were associated with lower nonper-CHR-P-sum-scores.

## Discussion

In this community study, we investigated the association of personality pathology and cognitive biases with CHR-P-symptom (i.e., UHR- and basic symptom) expression. In our findings, personality functioning was specifically associated with the presence of nonper-CHR-P-symptoms, with maladaptive personality traits not substantially contributing to the respective model. In contrast, cognitive biases significantly correlated with both the presence and severity of CHR-P-symptoms, showing a differential relationship to per- and nonper-CHR-P-symptoms.

Further, exploring the association between psychopathology and socio-occupational functioning with CHR-P-symptom expression, we found a positive association across models between more axis-I diagnoses and the likelihood of CHR-P-symptoms, while socio-occupational functioning was negatively associated with nonper-CHR-P-symptom likelihood only. The implications of our findings and our exploratory analyses involving personality functioning elements and individual cognitive biases will be discussed below.

### Personality functioning: Connections with nonper-CHR-P-symptoms

Overall, our results suggest a specific association between personality functioning impairment and a greater likelihood of nonper-CHR-P-symptoms, providing preliminary indications that the reported robust link between nonper-CHR-P- (especially UHR-) symptoms and impairment in psychosocial functioning [9] might extend to include personality functioning impairment. Conversely, we found no significant association between personality functioning impairment and either overall CHR-P-symptoms or per-CHR-P-symptoms. These findings highlight the need to further investigate the differential associations between personality functioning impairment and different categories of CHR-P-symptoms, for example, using data where rates of per- and nonper-CHR-P-symptoms allow for direct group comparison (see [9]). Considering (i) the established hypothesis linking nonper-CHR-P-symptoms to later-stage brain/cognitive maturation processes involving frontal regions [10] and (ii) existing evidence on frontal region activation in self- and other-referential processing relevant to personality functioning [89], future research should explore developmental and neurobiological correlates that might underlie the connection between nonper-CHR-P-symptoms and personality functioning in our study. Moreover, as negative CHR-P-symptoms were not assessed in the BEAR and BEARS-Kid studies, they were not considered in the current analysis. However, previous research has highlighted differential associations between personality pathology and positive versus negative subclinical psychotic symptoms [52], suggesting that some aspects of the relationship between personality functioning and nonper-, or even per- and overall CHR-P-symptoms, might have been masked in our analysis.

Additionally, our exploratory analysis indicated that the association between higher personality functioning impairment and greater likelihood of nonper-CHR-P-symptoms might particularly concern impairments in identity and self-direction (i.e., self-functioning). In our analysis, the nonper-CHR-P-sum-score predominantly consists of cognitive basic symptoms, which then likely weighed more on the statistical analyses than their UHR-symptom counterparts. Since basic symptoms are subjective disturbances, involving changes in mental processes that are immediately perceived to be distinct from those familiar to the self, they are by definition related to the self [40, 90, 91]. In turn, this close association with the self might then help explain the link between nonper-CHR-P-symptoms and personality functioning in our results. Moreover, this finding aligns with research connecting deficits in the corresponding personality functioning facets (e.g., self-others boundaries, emotional regulation abilities, self-esteem, productive self-reflection) to CHR-P-symptom expression and course [27, 40, 45, 46], although specific evidence on nonper-CHR-P-symptoms is lacking. In contrast, our finding of an association between higher impairments in identity and lower severity of nonper-CHR-P-symptoms seems incoherent with this reasoning. Possibly indicating a more complex relationship between identity and nonper-CHR-P-symptom expression, this warrants investigation beyond the scope of our cross-sectional study, under consideration of potential intervening factors, such as identity formation processes or positive resources buffering against nonper-CHR-P-symptom severity [5, 92, 93]. Speculatively, identity impairment might serve as a vulnerability factor for nonper-CHR-P-symptoms in their “trait-like,” “as-usual” manifestation, reflecting long-standing patterns less directly related to burden and psychosis risk [3,63]. Several other explanations for this finding, including Type I error, are also possible and should be rigorously tested in future studies. Finally, including maladaptive personality traits as a predictor of nonper-CHR-P-symptom expression did not improve this model. As our study design was guided by the AMPD, we only considered maladaptive personality traits when personality functioning showed a significant association to CHR-P-symptom expression, that is, only for nonper-CHR-P-symptoms. Therefore, while our work provides some support to the hypothesis that overarching features of personality, such as personality functioning, might be more closely associated with CHR-P expression than maladaptive traits [39], a better comprehension of their role should be pursued in future research, including all categories of CHR-P-symptoms as well as clinical samples.

### Cognitive biases: Unpacking complex associations

As a whole, more severe cognitive biases showed an association with both higher likelihood and severity of CHR-P-symptoms. Previous research has described a longitudinal link between cognitive biases and CHR-P-symptoms, proposing that cognitive biases might become a stable cognitive functioning feature, predisposing individuals to developing CHR-P-symptoms [94–96]. Furthermore, literature indicates that cognitive biases impact on multiple levels of perception, information processing, and related emotional reactions (e.g., worry), potentially interacting with stress responses that influence CHR-P-symptom severity [95, 97, 98]. While this reasoning aligns with our findings, we cannot disentangle whether (more severe) cognitive biases might be a consequence or a vulnerability/exacerbating factor of CHR-P-symptoms using our cross-sectional data [95, 99]. Addressing this question in longitudinal research might both expand our understanding of CHR-P-symptom expression and inform preventive interventions. Moreover, in our exploratory analysis, more severe dichotomous thinking, emotional reasoning, and catastrophizing were associated with higher likelihood of CHR-P-symptoms, with the first two also correlating with higher CHR-P-symptom severity. Consistent with existing data linking these cognitive biases to the presence and severity of subclinical positive symptoms in healthy individuals [100–102], these findings suggest that future research should explore their specific relevance to CHR-P-symptom expression in the community.

Furthermore, more severe cognitive biases were associated with higher likelihood of per- and severity of nonper-CHR-P-symptoms. Although our cross-sectional design precludes testing for directionality, the differential associations in our findings might reflect distinct underlying mechanisms and should be explored in future longitudinal studies. Based on our results, we might speculate that the predisposing function of cognitive biases for the development of CHR-P-symptoms is more closely related to per-CHR-P-symptoms and the connected earlier-stage maturation processes, while the impact of cognitive biases on CHR-P-symptoms rather concerns nonper-CHR-P-symptoms and the relative later-stage development processes [5, 6, 96, 102]. However, we wish to reiterate that this interpretation exceeds the scope of our study, and should only exemplify how our preliminary findings might help structuring hypotheses on the relationship between cognitive biases and per- versus nonper-CHR-P-symptoms, to then be tested elsewhere. Further, considering individual cognitive biases, the severity of dichotomous thinking and emotional reasoning was associated with increased likelihood of per-CHR-P-symptoms, consistent with previous findings in individuals with subclinical auditory hallucinations [100]. Similarly, we found an association of more severe dichotomous thinking and emotional reasoning with higher severity of nonper-CHR-P-symptoms, aligning with existing evidence on delusions [101]. Additionally, higher catastrophizing was associated with higher likelihood of nonper-CHR-P-symptoms, and higher intentionalizing with higher severity of nonper-CHR-P-symptoms. This reflected existing evidence on a link between catastrophizing and a higher likelihood of delusion presence and between intentionalizing and greater delusion severity [101]. Interestingly, higher intentionalizing correlated with less severe per-CHR-P-symptoms, suggesting a more complex relationship. This association might be influenced by the fact that, while evidence linked intentionalizing to perceptive symptoms via (the emotional component of) hallucinations [103], our per-CHR-P-sum-score predominantly consisted of basic symptoms. As this is true for all sum-scores, and evidence regarding the relationship between cognitive biases and basic symptoms is currently lacking, our results should overall be interpreted with caution and further investigated, especially considering our cross-sectional, explorative design. Offering an additional explanation for their correlation in our analyses, cognitive biases and (cognitive) basic symptoms both refer to aspects of cognitive functioning and thus, might have a reciprocal influence. Nonetheless, the two concepts are clearly distinct, with cognitive biases operating on the higher-level cognitive process of interpretation, which becomes systematically negatively distorted [52], whereas basic symptoms represent qualitative distortions in lower-level cognitive processes, like attention or concentration [4]. Finally, jumping to conclusions was the only cognitive bias for which severity was not associated with CHR-P-symptom expression. This aligns with indications that its influence might be specific to schizophrenia and active psychotic symptoms [56, 102, 104, 105]. Additionally, self-reporting on cognitive biases, and specifically on jumping to conclusions, might be skewed by factors like metacognitive awareness, which might lead community samples to report lower rates of jumping to conclusions (e.g., for reasons of social desirability) when compared to individuals with psychosis, whose metacognitive awareness might already be impaired. Overall, putting our results into perspective, previous research proposed that a general distorted thinking style (CBQp-sum-score) might be more clinically relevant than individual cognitive biases, for which evidence of distinct underlying distorted cognitive processes is inconsistent [56, 73, 94, 95].

### Psychopathology, functioning, and socio-demographics

In our analyses, current presence of more axis-I-diagnoses was associated with greater likelihood of CHR-P, per-CHR-P and nonper-CHR-P-symptoms, aligning with copious evidence of high comorbidity rates in CHR-P samples [18]. Further, lower socio-occupational functioning was associated with higher likelihood of nonper-CHR-P-symptoms, consistent with data on the close connection of especially non-perceptive UHR-symptoms with impaired functioning [9, 10, 17]. Moreover, findings of a significant link between age and overall CHR-P−/per-CHR-P-, but not nonper-CHR-P-, symptom severity are consistent with literature, but developmental implications cannot be drawn from our cross-sectional analyses [9, 10]. Finally, the link between female sex and higher CHR-P-symptom severity joins inconclusive evidence about sex effects on CHR-P expression [5]. Thus, findings involving age and sex require further investigation in future studies.

### Strengths and limitations

Next to the clear strengths of our study including the innovative focus on personality functioning in relation to CHR-P-symptoms and cognitive biases, and the large community sample, some limitations should be considered. As mentioned, no directionality can be inferred from our cross-sectional data, although, given the predominantly trait-like nature of cognitive biases and personality characteristics [29, 45], it seems plausible that they precede the state-like CHR-P-symptoms [106]. Further, in our exploratory analysis, we included individual cognitive biases and personality functioning elements separately in the relevant models to avoid multicollinearity, favored by high correlations between the subscales; this, however, also prevented examination of their interplay. Moreover, while we partially corrected for this in the outcomes variables by choosing to employ ZIP models, the low levels of impairment and pathology in our sample may restrict generalizability to other populations, as statistical power to detect associations within these limited ranges may be reduced. Additionally, data on negative, general, and disorganization SIPS-symptom scales, which might add more context to our findings [52], were not available to us, as assessments in the BEAR and BEARS-Kid studies focused on criteria-relevant UHR- and basic symptoms. Finally, as our sum-scores combine both basic symptoms and UHR-symptoms, the contributions of procedural versus content-related thought disorders are not discernible in our findings.

### Conclusion and future directions

The present study offers initial evidence on the intricate associations between personality functioning, cognitive biases, and CHR-P-symptomatology. First, nuanced associations of personality functioning, particularly identity and self-direction, with nonper-CHR-P manifestations emerged, alongside first indications of their relevance beyond maladaptive traits or personality disorders. Second, consistent with previous clinical studies [56], cognitive biases, and especially dichotomous thinking, emotional reasoning, and catastrophizing, arise as promising targets for future research on prevention through their association with CHR-P-symptoms likelihood and severity. Finally, our results support previous evidence on connections between nonper-CHR-P-symptoms and functioning impairment, as well as overall CHR-P expression and psychopathology [18]. Future longitudinal studies should test the associations in our findings and further investigate the complex interactions of personality pathology, psychosis risk, their related burden, and possible developmental implications, to extend our understanding of CHR-P-symptomatology.

## Supporting information

Rinaldi et al. supplementary material Rinaldi et al. supplementary material

## Data Availability

Data can be made available on request via the corresponding author (C.M.).

## Supplementary material

The supplementary material for this article can be found at http://doi.org/10.1192/j.eurpsy.2024.1812.
