# Supplementary material for: Investigating the associations between personality functioning, cognitive biases, and (non-)perceptive clinical high-risk symptoms of psychosis in the community
Source: Eur Psychiatry. 2025 Jan 22;68(1):e13. doi: 10.1192/j.eurpsy.2024.1812 (PMC11822964; doi:10.1192/j.eurpsy.2024.1812)
Supplement: Rinaldi et al. supplementary material [file S0924933824018121sup001.docx]

**Supplementary Material to:**

**Investigating the associations between personality functioning, cognitive biases, and (non-)perceptive clinical high-risk symptoms of psychosis in the community**

G. Rinaldi, S. Lerch, F. Schultze-Lutter, S.J. Schmidt, M. Cavelti, M. Kaess, C. Michel

**Contents : 1 eText, 1 eFigure, 7 eTables**

[eText 1*:* Second follow-up of the BEAR and the BEARS-Kid studies – study design and recruitment 2](#_Toc182937922)

[eFigure 1: The current sample 4](#_Toc182937923)

[eTable 1: Clinical high-risk symptoms and criteria of first-episode psychosis 5](#_Toc182937924)

[eTable 2: The Alternative Model of Personality Disorders in DSM-5 7](#_Toc182937925)

[eTable 3: Comparison of Poisson versus Zero-inflated Poisson (ZIP) models by AIC 9](#_Toc182937926)

[eTable 4*:* CHR-P-symptoms ZIP model, including non-significant predictors 9](#_Toc182937927)

[eTable 5*:* per-CHR-P-symptoms ZIP model, including non-significant predictors 10](#_Toc182937928)

[eTable 6*:* nonper-CHR-P-symptoms ZIP model, including non-significant predictors 10](#_Toc182937929)

[eTable 7*:* nonper-CHR-P-symptoms ZIP model, including PID-5-sumscore 11](#_Toc182937930)

[References 12](#_Toc182937931)

## eText 1*:* Second follow-up of the BEAR and the BEARS-Kid studies – study design and recruitment

Data analyzed in the current study pertains to the second follow-up assessment (ethics ID: 2020-02856) of the ‘Bern Epidemiological At-Risk’ (BEAR) and the ‘Bi-national Evaluation of At-Risk Symptoms in children and adolescents’ (BEARS-Kid) studies [1–3].

At baseline, participants in the two community studies were randomly selected from the population registry of Canton Bern, Switzerland, among about 384,000 persons in the targeted age range. Using a stratified sampling method, two representative samples of eligible residents aged 16-40 (BEAR, *N*=4,471, response rate: 63.4%) and 8-17 years (BEARS-Kid, *N*=980, response rate 32.6%) were thus obtained.

Selection of the first age range was based on the documented high frequency of psychotic experiences during this period and its consequent relevance for studying psychosis risk [4,5]. The second age range was chosen to explore the associations between neurodevelopment and CHR-P-symptoms, which are reported to be more frequent but less clinically significant in children and adolescents [6].

For both studies, inclusion criteria at baseline were main residence in Canton Bern and an available telephone number. Exclusion criteria, leading to premature interruption of the interview, were insufficient German, French, or English skills and a lifetime diagnosis of psychosis.

At baseline and previous follow-up assessments, participants in the BEARS-Kid study and their parents provided written informed assent/consent prior to face-to-face assessment, while, for the BEAR-study, participation in the telephone interview constituting the main assessment was equated to informed consent. Before data collection began, a feasibility study tested the reliability of the telephone assessment, reporting excellent (78-100%) concordance rates between telephone and face-to-face interviews, thus suggesting that merging data from the two studies would not introduce a systematic bias [7].

In both studies and for all time points, first telephone contact was attempted two weeks after sending a short information letter on study aims and procedure, as well as voluntariness of participation and anonymization of collected data used for scientific purposes.

At baseline, 2,683 participants were interviewed in the BEAR and 235 in the BEARS-Kid study, with about a quarter of them reporting CHR-P-symptoms and 2.4% meeting CHR-P criteria. For the first three-year follow-up of the BEAR-study, 1,263 participants were sampled (response rate 66.4%). Out of the 834 who were interviewed, 5 (0.5%) had converted to psychosis, all of whom had presented with CHR-P-symptoms/criteria at baseline (*N*=434). By the first two-year follow-up of the BEARS-Kid study (*N*=195, dropout rate 11%), one person who had reported CHR-P-symptoms at baseline had converted to psychosis. For the second ten-year-follow up, the two samples were merged (*N*=1,278), excluding both converters and those who did not give permission to be recontacted after the first follow-up. With data collection drawing to a close, 846 participants have been interviewed (response rate 85.5%), 2 of which have converted to psychosis. At baseline and second follow-up, there were no significant differences in age or sex between those who participated and those who did not (W=49,042, p=0.19; χ²=0.63, p=0.43), indicating that selection effects involving these key descriptive variables are unlikely. However, the sub-sample used in the current study seems to show a selection bias towards female, highly educated, and functionally unimpaired individuals. Two factors likely contributed to this bias. First, participation in the add-on study, which was an inclusion criterion for the present work, may have attracted individuals who were more motivated, had greater availability, or were more engaged with research – characteristics that might correlate with higher education levels and better functioning. Second, data collection for the second follow-up of the BEAR and BEARS-Kid studies was incomplete at the time of analysis. Individuals who had not yet been contacted may differ in socio-demographic characteristics due to logistical challenges, such as availability during specific calling hours or contact issues. These factors may inadvertently exclude certain populations (e.g., those with lower socioeconomic status or more demanding work schedules). Thus, while the original sample aimed to be representative, the sub-sample may not fully reflect the socio-demographic diversity of the Bernese community.

In the second follow-up, the same recruitment and assessment procedures of previous time points applied, with the exception that all interviews were conducted on the phone. Minors, who were all 14 years old and upwards, provided written informed consent/assent, and, for participants younger than 16, written informed consent by a parent was also required. This time, however, BEARS-Kid participants over 18 gave informed consent verbally by participating in the telephone interview. Additionally, German speaking participants who provided an *ad hoc* informed consent during the interview filled out add-on questionnaires online or, if they especially requested it, on paper. To enhance response rates, participants who did not fill out the questionnaires received a first e-mail reminder after three weeks, then regular reminders about once a year.

For both studies and all time points, interviews were conducted by clinical psychologists who participated in a three-month intensive training and weekly supervisions with F. Schultze-Lutter and/or C. Michel, ensuring assessment quality [8].

## eFigure 1: The current sample


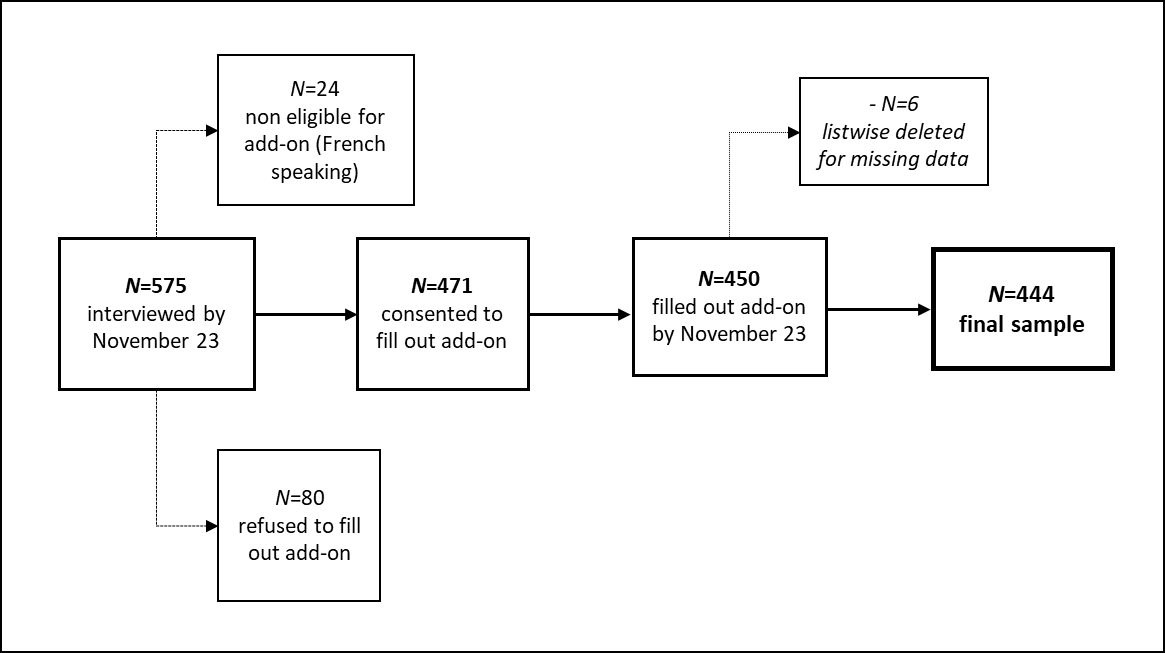


## eTable 1: Clinical high-risk symptoms and criteria of first-episode psychosis

| **Ultra-high risk (UHR) criteria** according to the SIPS |
| --- |
| A. ‘Brief Intermittent Psychotic Symptoms’ (BIPS)  ⮊ At least any 1 of the following SIPS P-items scored 6 ‘severe and psychotic’   - P1 Unusual Thought Content / Delusional Ideas - P2 Suspiciousness / Persecutory Ideas - P3 Grandiose Ideas - P4 Perceptual Abnormalities / Hallucinations - P5 Disorganized Communication   ⮊ First appearance in the past three months  ⮊ Present for at least several minutes per day at a frequency of at least once per month but less than 7 days |
| B. ‘Attenuated Positive Symptoms’ (APS)  ⮊ At least any 1 of the following SIPS P-items scored 3 ‘moderate’ to 5 ‘severe but not psychotic’   - P1 Unusual Thought Content / Delusional Ideas - P2 Suspiciousness / Persecutory Ideas - P3 Grandiose Ideas - P4 Perceptual Abnormalities / Hallucinations - P5 Disorganized Communication   ⮊ First appearance within the past year or current rating one or more scale points higher compared to 12 months ago  ⮊ Symptoms have occurred at an average frequency of at least once per week in the past month |
| C. ‘Genetic Risk and Deterioration’ Syndrome  (1) Patient meets criteria for Schizotypal Personality Disorder according to SIPS  (2) Patient has 1^st^ degree relative with a psychotic disorder  (3) Patient has experienced >30% drop in global assessment of functioning (GAF) score over the last month compared to 12 months ago  ⮊ [1 and 3] or [2 and 3] or all are met. |
| **Basic symptom criteria** |
| Risk criterion ‘Cognitive-Perceptive Basic Symptoms’ (COPER)  ⮊ At least any 1 of the following basic symptoms with a SPI-A score of ≥3 within the last 3 months:   - Thought interference - Thought perseveration - Thought pressure - Thought blockages - Disturbance of receptive speech - Decreased ability to discriminate between ideas and perception, fantasy and true memories - Unstable ideas of reference - Derealization - Visual perception disturbances (excluding hypersensitivity to light or blurred vision) - Acoustic perception disturbances (excluding hypersensitivity to sounds)   ⮊ First occurrence ≥12 months ago |
| Risk criterion ‘Cognitive Disturbances’ (COGDIS)  ⮊ At least any 2 of the following basic symptoms with a SPI-A score of ≥3 within the last 3 months:   - Inability to divide attention - Thought interference - Thought pressure - Thought blockages - Disturbance of receptive speech - Disturbance of expressive speech - Unstable ideas of reference - Disturbances of abstract thinking - Captivation of attention by details of the visual field |

## eTable 2: The Alternative Model of Personality Disorders in DSM-5

| **Diagnostic criteria for personality disorders in the AMPD** |
| --- |
| **Criterion A.** Moderate or greater impairment (i.e., a score of 2 or more) in personality functioning, manifesting in difficulties in two or more of the following four areas.  *“Self-functioning:*   1. Identity: Experience of oneself as unique, with clear boundaries between self and others; stability of self-esteem and accuracy of self-appraisal; capacity for, and ability to regulate, a range of emotional experience. 2. Self-direction: “Pursuit of coherent and meaningful short-term and life goals; utilization of constructive and prosocial internal standards of behavior; ability to self-reflect productively.   *Interpersonal functioning:*   1. Empathy: Comprehension and appreciation of others' experiences and motivations; tolerance of differing perspectives; understanding the effects of one's own behavior on others. 2. Intimacy: Depth and duration of connection with others; desire and capacity for closeness; mutuality of regard reflected in interpersonal behavior.”   (DSM-5, Section III, p. 762, table 1; [9]) |
| **Criterion B.** Maladaptive personality traits, organized in five domains, assessed dimensionally and each encompassing 25 facets.   - “Negative Affectivity (vs. Emotional Stability): Frequent and intense experiences of high levels of a wide range of negative emotions (e.g., anxiety, depression, guilt/ shame, worry, anger) and their behavioral (e.g., self-harm) and interpersonal (e.g., dependency) manifestations. - Detachment (vs. Extraversion): Avoidance of socioemotional experience, including both withdrawal from interpersonal interactions (ranging from casual, daily interactions to friendships to intimate relationships) and restricted affective experience and expression, particularly limited hedonic capacity. - Antagonism (vs. Agreeableness): Behaviors that put the individual at odds with other people, including an exaggerated sense of self-importance and a concomitant expectation of special treatment, as well as a callous antipathy toward others, encompassing both an unawareness of others' needs and feelings and a readiness to use others in the service of self-enhancement. - Disinhibition (vs. Conscientiousness) Orientation toward immediate gratification, leading to impulsive behavior driven by current thoughts, feelings, and external stimuli, without regard for past learning or consideration of future consequences. - Psychoticism (vs. Lucidity) Exhibiting a wide range of culturally incongruent odd, eccentric, or unusual behaviors and cognitions, including both process (e.g., perception, dissociation) and content (e.g., beliefs).”   (DSM-5, Section III, p. 762; [9]) |
| **Criteria C and D.** Relative pervasiveness and stability of impairments in personality functioning and pathological personality traits across personal and social contexts. |
| **Criteria E, F, and G:** Differential diagnoses, i.e., alternative explanations for personality pathology, such as other mental disorders, effects of a substance or medical condition, normal developmental stages, socio-cultural environment. |

## eTable 3: Comparison of Poisson versus Zero-inflated Poisson (ZIP) models by AIC

| eTable 3 AIC values (df), Poisson versus ZIP model | | | |
| --- | --- | --- | --- |
| Outcome | **Poisson model** | **ZlP model** |  |
| CHR-P-symptoms | 1098.04 (8) | 815.06 (16) | |
| nonper-CHR-P-symptoms | 808.79 (8) | 606.48 (16) |  |
| per-CHR-P-symptoms | 543.68 (8) | 455.93 (16) | |

*Note:* AIC: Akaike Information Criterion (lower value indicates better fit); df: degrees of freedom; CHR-P: clinical high-risk of psychosis; per-CHR-P: perceptive CHR-P; nonper-CHR-P: non-perceptive CHR-P.

## eTable 4*:* CHR-P-symptoms ZIP model, including non-significant predictors

| **eTable 4** Poisson regression (β) and logistic regression (γ) coefficients | | | | |
| --- | --- | --- | --- | --- |
|  | **Count model** | | **Zero-inflated model** | |
| **Predictor/covariate** | ***β±sd*** | ***p*** | ***γ±sd*** | ***p*** |
| Psychopathology | 0.07±0.04 | 0.10 | -0.70±0.19 | <.001 |
| Personality functioning | -0.12±0.07 | 0.10 | -0.30±0.16 | 0.05 |
| Cognitive biases | 0.20±0.07 | <.05 | -0.41±0.15 | <.05 |
| Socio-occupational functioning | 0.01±0.06 | 0.90 | 0.10±0.14 | 0.49 |
| Age | -0.03±0.01 | <.001 | -0.01±0.02 | 0.38 |
| Sex | 0.32±0.16 | 0.05 | -0.38±0.29 | 0.19 |
| Education level | 0.02±0.10 | 0.86 | 0.46±0.33 | 0.15 |

*Note:* CHR-P: clinical high-risk of psychosis; sd: standard deviation.
The β coefficients refer to the association between the predictor/covariate value and the outcome value (i.e., severity); thus, negative coefficients indicate lower outcome values when predictor/covariate values are higher, while positive coefficients indicate higher outcome values when predictor/covariate values are higher. The γ coefficients refer to the association between the predictor/covariate value and the likelihood of the outcome value being zero; thus, negative coefficients indicate lower likelihood of the outcome value being zero when predictor/covariate values are higher, while positive coefficients indicate higher likelihood of the outcome value being zero when predictor/covariate values are higher.

## eTable 5*:* per-CHR-P-symptoms ZIP model, including non-significant predictors

| **eTable 5** Poisson regression (β) and logistic regression (γ) coefficients | | | | |
| --- | --- | --- | --- | --- |
|  | **Count model** | | **Zero-inflated model** | |
| **Predictor/covariate** | ***β±sd*** | ***p*** | ***γ±sd*** | ***p*** |
| Psychopathology | -0.03±0.06 | 0.63 | -0.76±0.18 | <.001 |
| Personality functioning | -0.03±0.10 | 0.80 | 0.05±0.17 | 0.77 |
| Cognitive biases | -0.03±0.10 | 0.78 | -0.52±0.18 | <.05 |
| Socio-occupational functioning | 0.01±0.09 | 0.93 | -0.06±0.17 | 0.73 |
| Age | -0.02±0.01 | <.05 | -0.01±0.02 | 0.71 |
| Sex | 0.40±0.23 | 0.08 | -0.09±0.33 | 0.78 |
| Education level | -0.08±0.18 | 0.67 | 0.06±0.30 | 0.84 |

*Note:* per-CHR-P: perceptive CHR-P; sd: standard deviation.
The β coefficients refer to the association between the predictor/covariate value and the outcome value (i.e., severity); thus, negative coefficients indicate lower outcome values when predictor/covariate values are higher, while positive coefficients indicate higher outcome values when predictor/covariate values are higher. The γ coefficients refer to the association between the predictor/covariate value and the likelihood of the outcome value being zero; thus, negative coefficients indicate lower likelihood of the outcome value being zero when predictor/covariate values are higher, while positive coefficients indicate higher likelihood of the outcome value being zero when predictor/covariate values are higher.

## eTable 6*:* nonper-CHR-P-symptoms ZIP model, including non-significant predictors

| **eTable 6** Poisson regression (β) and logistic regression (γ) coefficients | | | | |
| --- | --- | --- | --- | --- |
|  | **Count model** | | **Zero-inflated model** | |
| **Predictor/covariate** | ***β±sd*** | ***p*** | ***γ±sd*** | ***p*** |
| Psychopathology | 0.06±0.07 | 0.36 | -0.76±0.28 | <.05 |
| Personality functioning | -0.14±0.12 | 0.26 | -0.64±0.26 | <.05 |
| Cognitive biases | 0.43±0.11 | <.001 | -0.19±0.22 | 0.39 |
| Socio-occupational functioning | 0.10±0.09 | 0.24 | 0.61±0.31 | <.05 |
| Age | -0.02±0.01 | 0.14 | 0.02±0.02 | 0.34 |
| Sex | 0.31±0.30 | 0.30 | -0.81±0.48 | 0.09 |
| Education level | -0.05±0.14 | 0.72 | 0.85±0.40 | <.05 |

*Note:* nonper-CHR-P: non-perceptive CHR-P; sd: standard deviation.
The β coefficients refer to the association between the predictor/covariate value and the outcome value (i.e., severity); thus, negative coefficients indicate lower outcome values when predictor/covariate values are higher, while positive coefficients indicate higher outcome values when predictor/covariate values are higher. The γ coefficients refer to the association between the predictor/covariate value and the likelihood of the outcome value being zero; thus, negative coefficients indicate lower likelihood of the outcome value being zero when predictor/covariate values are higher, while positive coefficients indicate higher likelihood of the outcome value being zero when predictor/covariate values are higher.

## eTable 7*:* nonper-CHR-P-symptoms ZIP model, including maladaptive personality traits (PID-5-sumscore)

| **eTable 7** Poisson regression (β) and logistic regression (γ) coefficients | | | | |
| --- | --- | --- | --- | --- |
|  | **Count model** | | **Zero-inflated model** | |
| **Predictor/covariate** | ***β±sd*** | ***p*** | ***γ±sd*** | ***p*** |
| Psychopathology | 0.06±0.07 | 0.38 | -0.71±0.27 | <.05 |
| Personality functioning | -0.22±0.18 | 0.23 | -0.28±0.37 | 0.46 |
| Cognitive biases | 0.42±0.11 | <.001 | -0.17±0.23 | 0.45 |
| Maladaptive personality traits | 0.12±0.17 | 0.46 | -0.48±0.33 | 0.15 |
| Socio-occupational functioning | 0.12±0.08 | 0.17 | 0.64±0.29 | <.05 |
| Age | -0.02±0.01 | 0.14 | 0.02±0.02 | 0.38 |
| Sex | 0.30±0.30 | 0.32 | -0.94±0.48 | 0.05 |
| Education level | -0.03±0.14 | 0.83 | 0.89±0.41 | <.05 |

*Note:* nonper-CHR-P: non-perceptive CHR-P; sd: standard deviation.
The β coefficients refer to the association between the predictor/covariate value and the outcome value (i.e., severity); thus, negative coefficients indicate lower outcome values when predictor/covariate values are higher, while positive coefficients indicate higher outcome values when predictor/covariate values are higher. The γ coefficients refer to the association between the predictor/covariate value and the likelihood of the outcome value being zero; thus, negative coefficients indicate lower likelihood of the outcome value being zero when predictor/covariate values are higher, while positive coefficients indicate higher likelihood of the outcome value being zero when predictor/covariate values are higher.

## References

[1] Schultze-Lutter F, Michel C, Ruhrmann S, Schimmelmann BG. Prevalence and clinical relevance of interview-assessed psychosis-risk symptoms in the young adult community. Psychol Med. 2017/09/11 ed. 2018;48(7):1167–78. https://doi.org/10.1017/S0033291717002586.

[2] Schultze-Lutter F, Ruhrmann S, Michel C, Kindler J, Schimmelmann BG, Schmidt SJ. Age effects on basic symptoms in the community: A route to gain new insight into the neurodevelopment of psychosis? Eur Arch Psychiatry Clin Neurosci. 2020 Apr 1;270(3):311–24. https://doi.org/10.1007/s00406-018-0949-4.

[3] Schultze-Lutter F, Schimmelmann BG, Michel C. Clinical high-risk of and conversion to psychosis in the community: A 3-year follow-up of a cohort study. Schizophr Res. 2021 Feb;228:616–8. https://doi.org/10.1016/j.schres.2020.11.032

[4] Kirkbride JB, Fearon P, Morgan C, Dazzan P, Morgan K, Tarrant J, et al. Heterogeneity in Incidence Rates of Schizophrenia and Other Psychotic Syndromes: Findings From the 3-Center ÆSOP Study. Arch Gen Psychiatry. 2006 Mar 1;63(3):250–8. https://doi.org/10.1001/archpsyc.63.3.250.

[5] McGrath JJ, Saha S, Al-Hamzawi AO, Alonso J, Andrade L, Borges G, et al. Age of Onset and Lifetime Projected Risk of Psychotic Experiences: Cross-National Data From the World Mental Health Survey. Schizophr Bull. 2016 Jul 1;42(4):933–41. https://doi.org/10.1093/schbul/sbw011.

[6] Schultze-Lutter F, Schimmelmann BG, Flückiger R, Michel C. Effects of age and sex on clinical high-risk for psychosis in the community. World J Psychiatry. 2020 May 19;10(5):101–24. https://doi.org/10.5498/wjp.v10.i5.101.

[7] Michel C, Schimmelmann BKE, Kupferschmid S, Siegwart M, Schultze-Lutter F. Reliability of telephone assessments of at-risk criteria of psychosis: a comparison to face-to-face interviews. Schizophr Res. 2014;153(1–3):251–3. https://doi.org/10.1016/j.schres.2014.01.025.

[8] Michel C, Schimmelmann BG, Schultze‐Lutter F. Demographic and clinical characteristics of diagnosed and non‐diagnosed psychotic disorders in the community. Early Interv Psychiatry. 2018;12(1):87–90. https://doi.org/10.1111/eip.12360.

[9] American Psychiatric Association. Diagnostic and Statistical Manual of Mental Disorders [Internet]. Fifth Edition. American Psychiatric Association; 2013 [accessed 2024 Feb 9]. https://doi.org/10.1176/appi.books.9780890425596.
